# Causal relationships between type 1 diabetes mellitus and Alzheimer’s disease and Parkinson’s disease: a bidirectional two-sample Mendelian randomization study

**DOI:** 10.1186/s40001-023-01628-z

**Published:** 2024-01-16

**Authors:** Chaofan Geng, Ke Meng, Bo Zhao, Xiaoduo Liu, Yi Tang

**Affiliations:** 1 Department of Neurology & Innovation Center for Neurological Disorders, Xuanwu Hospital, Capital Medical University, National Center for Neurological Disorders, 45 Changchun Street, Beijing, 100053 China; 2 Department of Neurology, Rongcheng People’s Hospital, The Affiliated Hospital of Jining Medical University, Weihai, China; 3 Neurodegenerative Laboratory of Ministry of Education of the People’s Republic of China, Beijing, China

**Keywords:** Type 1 diabetes mellitus, Mendelian randomization, Alzheimer’s disease, Parkinson’s disease

## Abstract

**Background:**

Previous compelling evidence suggests an association between Type 2 diabetes (T2D) and neurodegenerative diseases. However, it remains uncertain whether Type 1 diabetes mellitus (T1DM) exerts a causal influence on the risk of Alzheimer's disease (AD) and Parkinson's disease (PD). Consequently, this study employed a bidirectional two-sample Mendelian Randomization (MR) approach to investigate the causal relationship between T1DM and the genetic susceptibility to AD and PD.

**Methods:**

We utilized large-scale cohorts derived from publicly available genome-wide association study datasets involving European populations to perform MR analyses. The primary analytical method employed was the inverse-variance weighted (IVW) approach. Furthermore, sensitivity analyses, including assessments of heterogeneity and horizontal pleiotropy, were carried out using Cochran's Q, MR-Egger intercept, and MR-PRESSO tests to enhance the robustness of our conclusions.

**Results:**

Using the IVW-based method, the MR analysis indicated no significant association between genetically determined T1DM and AD (OR = 0.984, 95% CI: 0.958–1.011, *p* = 0.247). Conversely, T1DM appeared to be associated with a reduced risk of genetic susceptibility to PD (IVW: OR = 0.958, 95% CI: 0.928–0.989, *p* = 0.001). In the reverse direction, no evidence of reverse causality was observed between AD (OR = 1.010, 95% CI: 0.911–1.116, *p* = 0.881) or PD (OR = 1.164, 95% CI: 0.686–2.025, *p* = 0.5202) and T1DM. Additionally, our analysis found no indications of the results being influenced by horizontal pleiotropy.

**Conclusion:**

This MR study reveals that T1DM is associated with a reduced genetic susceptibility to PD, whereas no significant genetic susceptibility is observed between T1DM and AD. These findings suggest that T1DM may have a distinct role in the development of neurodegenerative diseases compared to T2D. Further investigations are warranted to elucidate the underlying mechanisms and provide a more comprehensive understanding of this relationship.

**Supplementary Information:**

The online version contains supplementary material available at 10.1186/s40001-023-01628-z.

## Introduction

Type 1 diabetes mellitus (T1DM) is an organ-specific autoimmune disorder influenced by a combination of genetic and environmental factors [1, 2]. As the disease progresses, autoimmune destruction of pancreatic β-cells intensifies, resulting in a decline or complete loss of their function. This leads to the manifestation of clinical symptoms of diabetes mellitus and a lifelong dependency on insulin therapy [3]. An emerging body of evidence indicates a close connection between insulin resistance and the pathogenesis of neurodegenerative conditions, including Alzheimer's disease (AD) and Parkinson's disease (PD) [4, 5]. T1DM has been postulated to expedite cognitive decline [6],with reports of elevated levels of phosphorylated Tau protein in T1DM patients [7], a factor associated with increased intracellular neurofibrillary tangle (NFT) formation in AD [6]. Furthermore, prior research has suggested that T1DM might act as a predisposing factor for PD [8].

However, it is important to note that the association between T1DM and the risk of developing AD and PD remains uncertain due to very limited studies and inconsistent findings. Whether T1DM itself causally contributes to the risk of AD and PD is still an unresolved question. Therefore, novel research approaches are essential to gain a comprehensive understanding of this association.

Recently, the utilization of large-scale genome-wide association study (GWAS) data and substantial sample sizes has introduced Mendelian Randomization (MR) as a powerful analytical method. MR employs genetic variants, typically single nucleotide polymorphisms (SNPs), as instrumental variables (IVs) to estimate the causal relationship between an exposure and a disease [9, 10]. It addresses issues of confounding and reverse causality more effectively, resembling the randomized controlled trial design due to the random assortment and combination of alleles during gamete formation [9], MR studies offer a higher level of evidence compared to observational studies [11]. In this study, we conducted a bidirectional two-sample MR analysis utilizing GWAS databases to systematically investigate the genetic causality between T1DM and the risk of AD and PD.

## Materials and methods

### Study design and MR assumptions

**Fig. 1:**
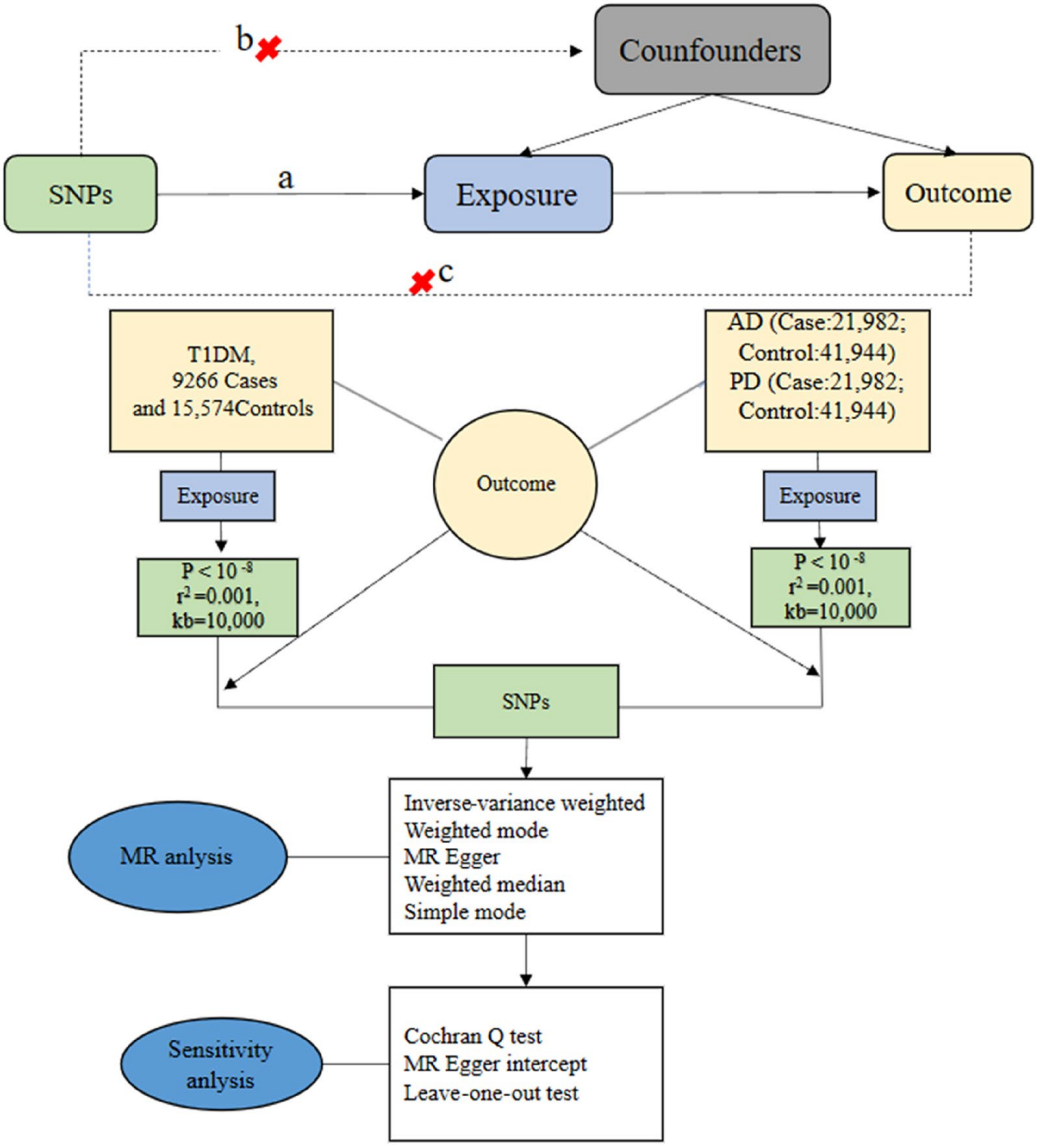
Flowchart of study. Assumption **a** IVs directly affect exposure; Assumption **b** IVs are not associated with confounders; Assumption **c** IVs influence risk of the outcome directly through the exposure

To investigate bidirectional associations between T1DM and AD as well as PD through MR studies, we applied three fundamental assumptions to genetic variants [12]: 1) the assumption of association, which states that SNPs are closely linked to the exposure; 2) the assumption of independence, implying that SNPs are free from confounders along the exposure-outcome pathway; and 3) the assumption of exclusivity, suggesting that SNPs exclusively influence the outcome through exposure and not via other pathways. Figure 1 provides an overview of our study design.

### Data source

We obtained aggregated statistics for T1DM in individuals of European descent from the European Bioinformatics Institute (EBI) database [13]. This dataset represents an extensive interdisciplinary and intercontinental resource, encompassing the largest and most up-to-date GWAS study of T1DM and comprising 9,266 cases and 15,574 controls [14].

For the identification of genetic variants associated with AD prevalence, we utilized the meta-analysis data from the International Genomics of Alzheimer's Project (IGAP) [15]. This dataset included a total of 63,926 subjects (21,982 AD cases and 41,944 healthy controls) of European origin, diagnosed with AD through autopsy or clinical diagnostic criteria. We analyzed data from a large-scale GWAS meta-analysis conducted by the International Parkinson's Disease Genomics Consortium (IPDGC) for PD phenotypes, which comprised 33,674 cases and 449,056 controls [16].

### Selection criteria for IVs

In accordance with the core assumptions of MR studies, we included SNPs with correlations satisfying *P* < 5 × 10^–8^ as instrumental variables by screening the GWAS data. To mitigate the impact of linkage disequilibrium (LD) on analysis results, we enforced the condition of *r*^2^ < 0.001 and window size = 10,000 kb [17]. To ensure robust associations between instrumental and endogenous variables and to prevent weak instrumental variable bias, we calculated *R*^2^ [*R*^2^ = 2 × EAF × (1 − EAF) × *b*^2^], representing the proportion of variation explained by instrumental variable SNPs, and the *F* statistic [*F* = *R*^2^ × (*N* − 2)/(1 − *R*^2^)], used to evaluate the strength of instrumental variables, for each SNP separately [18, 19]. Additionally, SNPs that were solely associated with the outcome through exposure were identified using the PhenoScanner (V2) database (http://www.phenoscanner.medschl.cam.ac.uk/).

### Mendelian randomization study and sensitivity analysis

In this MR study, we primarily employed the inverse-variance weighted (IVW) method to explore the causal relationship between T1DM and AD as well as PD. To ensure the robustness of our statistical findings, we conducted sensitivity analyses using both the weighted median (WM) and Mendelian randomization-egger regression (MR-Egger) based on Egger regression. The IVW method is considered the standard approach for MR pooled data [20], utilizing the Wald ratio method to estimate the causal effect for each included instrumental SNP, followed by a weighted pooled analysis [20]. The weighted median estimation method requires that at least 50% of the weights contributed by genetic variation are valid for statistical calculations [21]. MR-Egger regression identifies and corrects for multicollinearity, provided that the included instrumental variables satisfy the instrument strength independent of direct effect (INSIDE) assumption, which assumes independence between instrument-exposure and instrument-outcome associations [22]. Furthermore, weighted median [21] and maximum likelihood [23] methods were employed as complementary approaches to assess potential causality.

For sensitivity analyses, we calculated Cochran's Q statistic using both IVW and MR-Egger regression. A *P*-value > 0.05 indicates no significant heterogeneity. Additionally, we employed the leave-one-out method, systematically excluding each included SNP one by one, and generated forest plots. A *P*-value > 0.05 after excluding a SNP suggests that the SNP does not significantly affect the results [20]. To assess pleiotropy, we used both the intercept term of MR-Egger regression and the Mendelian randomization pleiotropy residual sum and outlier (MR-PRESSO) test for the included SNPs. In MR-Egger regression, an intercept trending towards zero indicates the absence of horizontal pleiotropy. The MR-PRESSO test calculates the degree of influence of included instrumental variables and assesses the effect size between exposure and outcome after removing outliers, thereby allowing a pre- and post- correction comparison of results [24]. In this MR analysis, odds ratio (OR) served as the effect value, and a 95% confidence interval (CI) was applied. Statistical significance was considered at *P* < 0.05. The R 4.0.3 software, along with the Two-Sample-MR [25] and MR-PRESSO [24] packages, were used for data processing and visualization.

## Results

### Results of the MR study

**Fig. 2:**
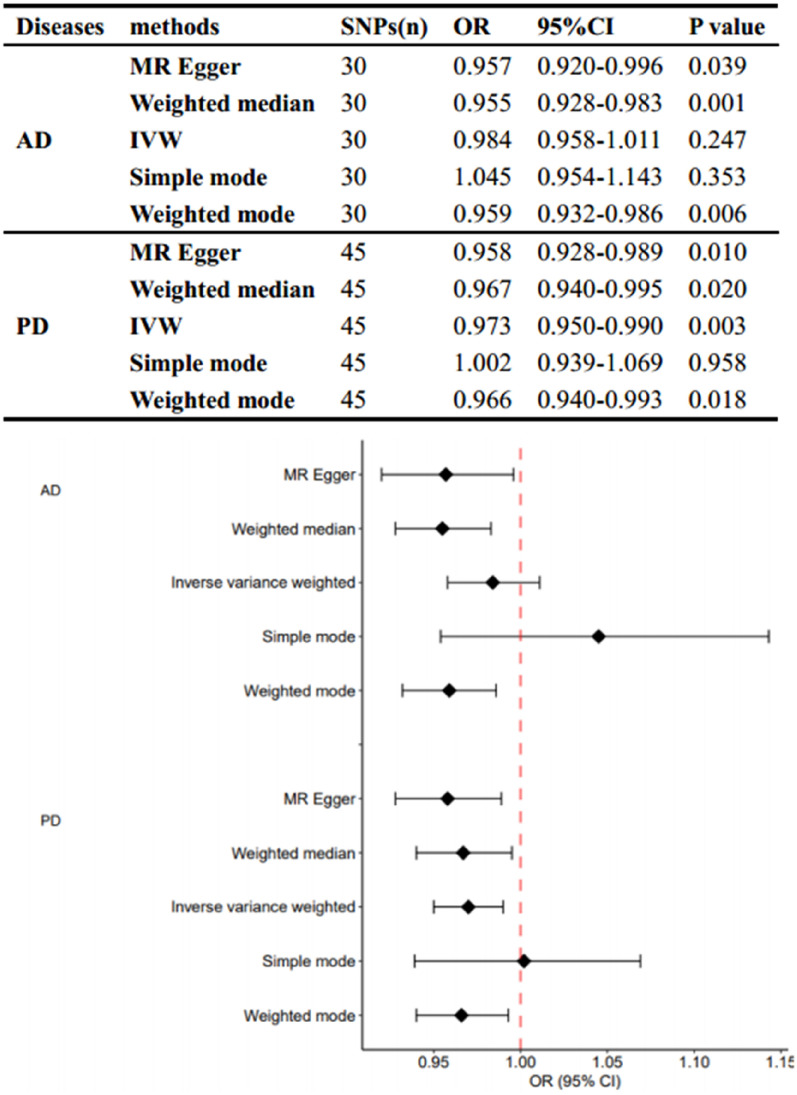
Results of MR analyses conducted to estimate potential associations between T1DM and risk of AD and PD. *OR* odds ratio, *CI* confidence interval, *MR* mendelian randomization, *IVW* Inverse variance weighted

The numbers of SNPs that were ultimately identified as the IVs in the different outcome datasets were 30 (AD) and 45 (PD), respectively. Using the IVW-based method, the MR analysis demonstrated no significant correlation between genetically determined T1DM and AD (OR = 0.984, 95% CI: 0.958–1.011, *p* = 0.247). However, it's important to note that the results from other methods differed (MR Egger: OR = 0.957, 95% CI: 0.920–0.996, *p* = 0.039; Weighted median: OR = 0.955, 95% CI: 0.928–0.983, *p* = 0.001; Weighted mode: OR = 0.959, 95% CI: 0.932–0.986, *p* = 0.006) (Fig. 2).

Conversely, the IVW model indicated that T1DM appeared to reduce the risk of genetic susceptibility to PD (OR = 0.958, 95% CI: 0.928–0.989, *p* = 0.001), a finding consistent with other methods (MR Egger: OR = 0.957, 95% CI: 0.920–0.996, *p* = 0.039; Weighted median: OR = 0.967, 95% CI: 0.940–0.995, p = 0.020; Weighted mode: OR = 0.966, 95% CI: 0.940–0.993, *p* = 0.018) (Fig. 2).

### Results of sensitivity analyses in the MR study

**Fig. 3.**
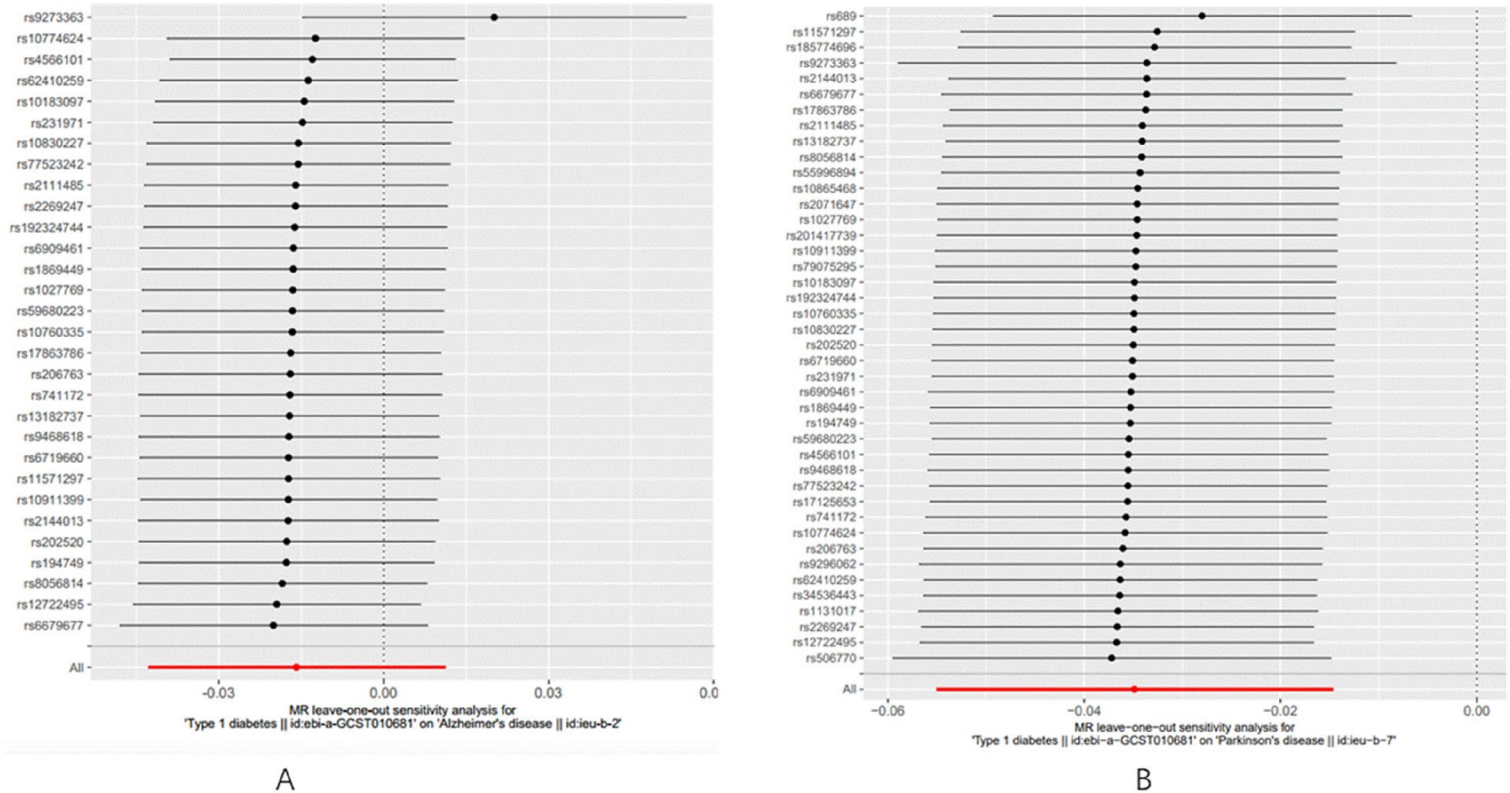
**A** MR leave-one-out analysis for T1DM and AD. **B** MR leave-one-out analysis for T1DM and PD

In the assessment of heterogeneity using Cochran’s Q test, significant heterogeneity was observed in the IVW and MR-Egger methods for AD (MR Egger: Q = 43.75, *p* = 0.029; IVW: Q = 48.92, *p* = 0.011), suggesting the presence of heterogeneity among SNPs. Consequently, a random-effects IVW model was employed to mitigate the impact of heterogeneity on AD in the MR study. The MR-Egger intercept found no evidence of horizontal pleiotropy (*p* > 0.05), indicating that horizontal pleiotropy did not significantly affect the study results. The leave-one-out sensitivity analysis further confirmed that no single SNP was driving the causal effect (Table 1, Fig. 3A).

**Table 1:** The results of heterogeneity and horizontal pleiotropy tests

Diseases	TIDM			
	Heterogeneity		Horizontal pleiotropy tests	
	MR-egger regression	IVW model	MR-egger intercept	MR PRESSO test
AD	0.029	0.011	0.079	NA
PD	0.300	0.298	0.713	0.305

*DTI * Townsend deprivation index,* IVW*, Inverse variance weight

**Fig. 4:**
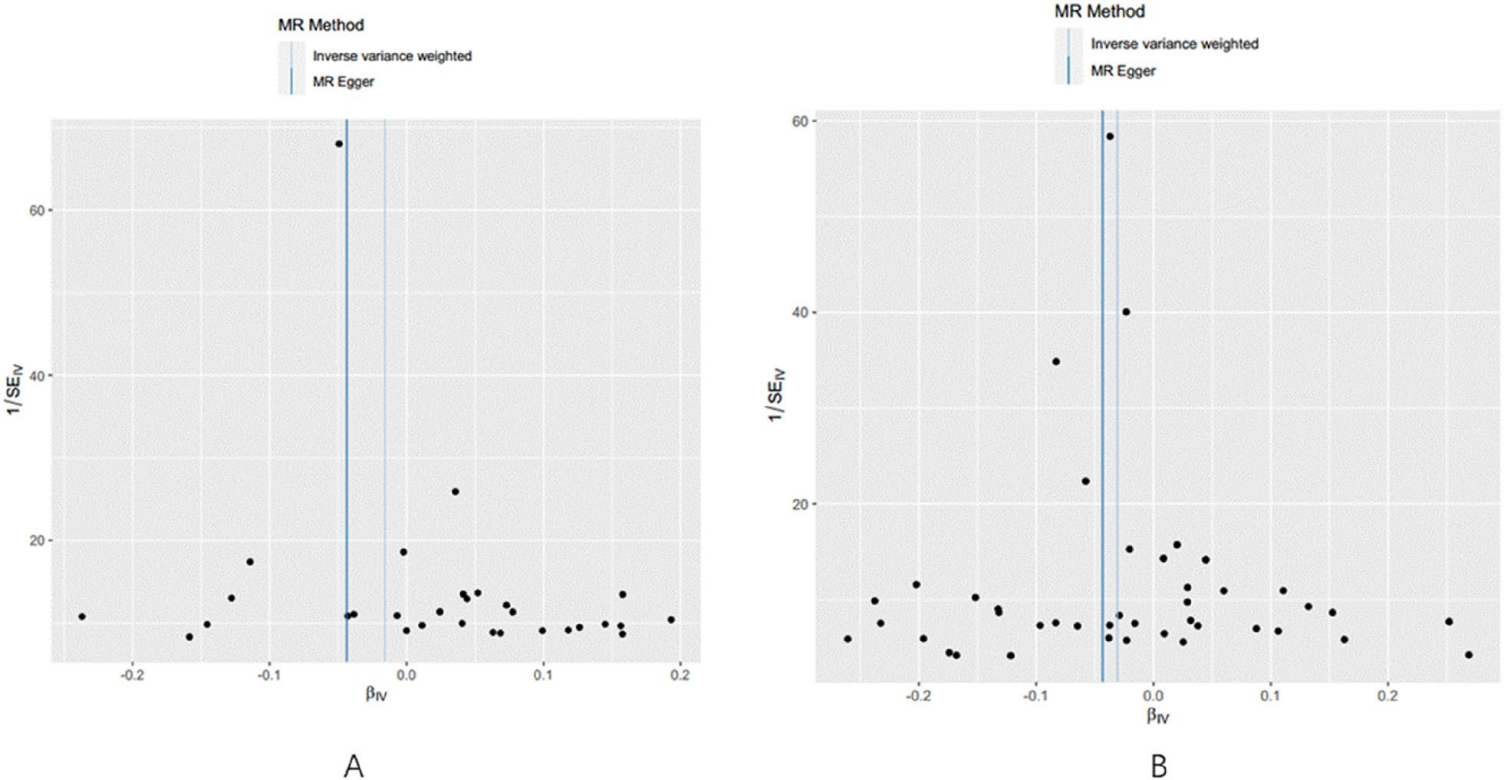
**A** Funnel plots of the association between T1DM and AD. **B** Funnel plots of the association between T1DM and PD. The dark blue line represents MR-Egger and the light blue line represents IVW

**Fig. 5:**
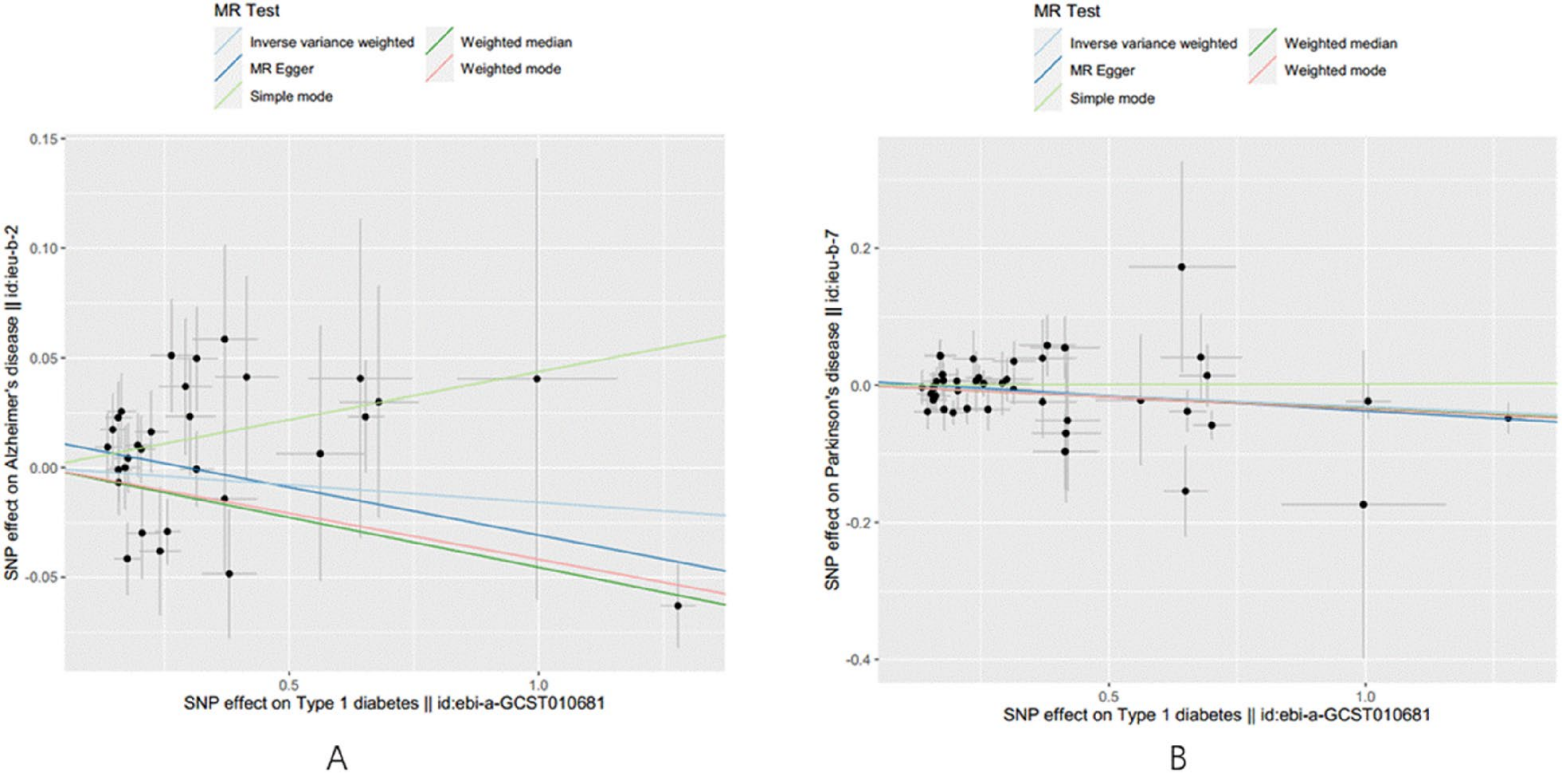
Scatter plot of genetic correlation between T1DM and AD and PD using five MR methods. **A** Evaluation the effect of T1DM on AD; **B** Evaluation the effect of T1DM on PD. *MR* Mendelian randomization

In contrast, for PD, there was no evidence of heterogeneity or horizontal pleiotropy (*p* > 0.05). The leave-one-out sensitivity analysis also indicated that no single SNP was exerting a dominant influence on the causal effect (Table 1, Fig. 3B). Additionally, funnel plots and scatter plots were used for visualization (Figs. 4 and 5).

### Results of reverse MR study and sensitivity analyses

**Fig. 6:**
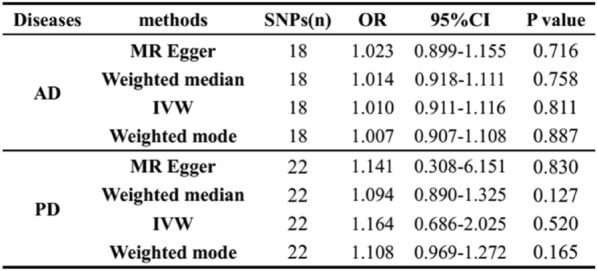
Results of the reverse MR study between AD or PD and T1DM. *OR* odds ratio, *CI* confidence interval, *MR* mendelian randomization, *IVW* Inverse variance weighted

In the reverse MR study, we identified 18 SNPs for the analysis of AD and 22 SNPs for the analysis of PD. Due to the presence of heterogeneity (MR Egger: Q = 28.83, *p* = 0.025; IVW: Q = 28.98, *p* = 0.034), the final reverse MR study did not establish reverse causality between AD and T1DM, based on the results of the random-effects IVW model (AD: OR = 1.010, 95% CI: 0.911–1.116, *p* = 0.881). The results were consistent across MR Egger, weighted median, and weighted mode methods (MR Egger: OR = 1.023, 95% CI: 0.899–1.155, *p* = 0.716; weighted median: OR = 1.023, 95% CI: 0.899–1.155, *p* = 0.716; weighted mode: OR = 1.007, 95% CI: *p* = 0.887) (Fig. 6). Moreover, there was no evidence of horizontal pleiotropy (MR-Egger intercept: *p* = 0.774) (Table 1).

Regarding PD, significant heterogeneity was observed (MR Egger: p < 0.001; IVW: *p* < 0.001). Using the random-effects IVW model, no reverse causality was found between PD and T1DM (OR = 1.164, 95% CI: 0.686–2.025, *p* = 0.5202). Similar results were obtained with MR Egger, weighted median, and weighted mode methods (MR Egger: OR = 1.141, 95% CI: 0.308–6.151, *p* = 0.830; weighted median: OR = 1.094, 95% CI: 0.890–1.325, *p* = 0.127; weighted mode: OR = 1.108, 95% CI: 0.969–1.272, *p* = 0.165). There was no evidence of horizontal pleiotropy (MR-Egger intercept: *p* = 0.972) (Fig. 6). More detailed results are presented in Additional file 1: Figs. S1–S6.

## Discussion

Previous epidemiological investigations have consistently linked type 2 diabetes mellitus (T2DM) with neurodegenerative disorders, including AD and PD [26]. For example, T2DM can lead to a significantly increased risk of AD and PD [27–29]. However, the relationship between T1DM and these neurodegenerative diseases has received less attention. In this study, we employed a bidirectional two-sample MR approach to assess the causal association between T1DM and AD and PD, aiming to provide valuable insights into these relationships and expand the knowledge in the field of diabetes and neurodegenerative disorders research. To our knowledge, this study represents the first application of bidirectional MR to investigate the genetic risk aspect of this association. Our findings suggest that there is no causal relationship between T1DM and AD. Conversely, genetic susceptibility to T1DM is associated with a reduced risk of PD. Additionally, the reverse MR analysis did not reveal any reverse causal links between AD or PD and T1DM.

### The connections between T1DM and AD

Extensive research has recently focused on the causal relationship between T2DM and AD [30]. Although previous studies focusing on the impact of T1DM-induced glycemic events on AD have been very limited, structural brain changes and cognitive impairments changes are common in individuals with T1DM, suggesting a potentially close relationship between T1DM and AD [31]. Additionally, glycemic control has been identified as a potentially modifiable factor in dementia prevention [32]. Several previous studies have explored the connection between T1DM and dementia risk, but the conclusions have been inconsistent [33, 34]. Notably, a longitudinal cohort study with a mean follow-up of 6.9 years found that elderly patients with T1DM, whether experiencing hypoglycemic (hazard ratio [HR] = 1.66, 95% CI: 1.09–2.53) or hyperglycemic events (HR = 6.20, 95% CI: 3.02–12.70), were at an increased risk of developing dementia [35]. This is particularly relevant as T1DM patients are more susceptible to hypoglycemic events compared to those with T2DM [35]. Additionally, hyperglycemic events in T1DM patients, have been shown to induce structural alterations with reduced gray matter density in the brain [36], including the posterior cingulate, hippocampus, and superior temporal gyrus, which are responsible for memory and language processing [30].

Nevertheless, due to limited studies, there is no convincing evidence supporting T1DM can increase the risk for AD. It is worth noting that in this study the focus was on assessing the causal relationship between T1DM and AD, in terms of the development and process of AD is beyond the scope of this specific study. Although we did not identify a genetic predisposition of T1DM to AD,

it is not contradictory to expound that T1DM can accelerate cognitive decline in AD patients. Studies have reported elevated levels of CSF Tau and Aβ_42_ in AD patients with T1DM [37]. Moreover, T1DM typically manifests at a young age, and patients require continuous insulin use from diagnosis [35]. Notably, CSF Aβ_42_ levels have been found to increase following peripheral insulin infusion, which may contribute to this phenomenon [38]. Moreover, hyperphosphorylation of Tau has been observed in the cortex and hippocampus of streptozotocin-induced T1DM mouse models; Additionally, mouse models of AD and T1DM have exhibited similar patterns of peripheral neuropathy [39]. High blood glucose levels can promote neuroinflammation and interrupt the integrity of the blood–brain barrier (BBB) [31]. Furthermore, increasing evidence has shown that the inflammatory response and BBB dysfunction generated by T1DM may contribute to microvascular damage and cardiovascular diseases [40], which involve in the pathogenesis of AD [30]. To sum up, the fact that T1DM and AD share similar biomarkers suggests that they may share similar pathologies in the process of disease and speed up cognitive decline instead of T1DM itself. Moreover, there is a lack of cohort studies on T1DM and the risk of AD, necessitating caution in interpreting this result. Further clinical studies and experimental evidence are warranted to elucidate this association.

### The connections between T1DM and PD

While there is mounting evidence linkingT2DM with PD [41, 42], and suggesting antidiabetic agents as a novel treatment for PD [5], less is known about the association between PD and T1DM. For PD, the changes in brain insulin resistance (BIR) and α-synuclein protein at synapses, as well as the dopaminergic loss in specific brain regions, eventually yield to the manifestation of the classic motor symptoms corresponding to the typical PD phenotype [43]. Moreover, dopaminergic neurons are particularly vulnerable to glucose toxicity due to their higher energy demands. Therefore, targeting insulin receptors in the central nervous system is crucial not only for individuals with diabetes but also for those with non-diabetic PD [42].

In our bidirectional two-sample MR study, we found that T1DM is associated with a decreased genetic susceptibility to PD, consistent with previous unidirectional MR research [44]. This methodology represents an advancement over previous research methods, enhancing the reliability of the conclusions. A drosophila model of T1DM has shown reduced levels of tyrosine hydroxylase in the brain, a typical PD-related phenotype [8], suggesting that T1DM might be a risk factor for developing PD. On the other hand, compared to T1DM, T2MD model may be more associated with PD and has been demonstrated that more suitable for studying metabolic disturbance, which acts an early risk factor for PD [31]. Notably, to the best of our knowledge, no cohort study has examined the association between T1DM and PD. Therefore, future studies should investigate this potential relationship with larger sample sizes.

There are some potential mechanisms may partly contribution to the difference patterns of PD in T1DM and T2DM. In contrast to autoimmune-mediated β-cell failure leading to absolute insulin deficiency in individuals with T1DM, there is no compelling evidence to support an autoimmune response in T2DM [1]. On one hand, the diagnosis of T1DM occurs at a younger age, persists throughout the lifespan, results in the loss of pancreatic beta-cell function in about 80% of the pancreas at the time of diagnosis, and will have been on continuous insulin use since diagnosis. The effect of long-term insulin use on movement disorders in PD has not been studied. On the other hand, the GWAS have been determined to strongly emphasize the genetic differences between T1DM and T2DM. All in all, although T1DM and T2DM share the common feature of β-cell failure, different types of diabetes may play different roles in PD.

Several factors may explain the reduced risk of PD in T1DM patients. Mitochondrial dysfunction and oxidative stress are well-established contributors to the pathophysiology of PD [45]. Recent studies have identified genetic overlaps between diabetes and PD [46], including genes such as *IGF2* and *MEG3*. *IGF2* has been shown to protect against oxidative and neuronal damage in cellular and mouse models of PD [47]. Increasing evidence suggests that *IGF2* may play neurotrophic and neuroprotective roles in various neurodegenerative diseases [48]. However, plasma *IGF2* levels have been reported as significantly lower in PD patients compared to healthy controls [49]. Similarly, *MEG3* expression was downregulated in PD patients [50], which can influence LRRK2 expression and regulate apoptosis in PD. These findings may be explained by disease heterogeneity or the presence of comorbidities, necessitating validation with larger sample sizes. The lysosomal-autophagy pathway has been proposed to explain the potentially protective effect of T1DM against PD [51], supported by genetic overlap between *CTSB* and *RAB7L1* in PD and T1DM [44]. Future research should explore additional potential mechanisms to further elucidate these findings.

### Advantages and limitations

This study offers several research advantages. Firstly, the application of the MR model effectively mitigated the influence of confounding variables and reverse causation, yielding reliable causal effect estimates, a notable improvement over standard observational studies [52]. Moreover, the utilization of a large-sample GWAS dataset significantly enhanced test efficacy when compared to small-sample models relying on individual data [20]. Secondly, the MR approach simultaneously controlled for instrumental variable errors related to both exposure and outcome, while also correcting for the bias introduced by LD among instrumental variables [20]. Lastly, bidirectional MR studies have the distinct advantage of effectively sidestepping reverse causation effects and minimizing residual confounding.

Nevertheless, there are several limitations in our study. Firstly, since all data were sourced from individuals of European descent, the results may not be generalizable to populations of different ethnic backgrounds. Secondly, due to the unavailability of gender- or age-stratified data in the GWAS datasets used, we were unable to assess whether the associations between T1DM and AD and PD differ across gender or age groups. Further research should explore these potential variations when stratified GWAS pooled data become accessible. Third, despite the comprehensive sensitivity analyses conducted to test MR study hypotheses, complete elimination of the possibility of horizontal pleiotropy among instrumental variables remains challenging. Lastly, differences in gene annotation analysis platforms across GWAS cohort studies may have contributed to the heterogeneity of this study. Heterogeneity was observed between instrumental variables concerning AD and T1DM, as indicated by the results of the heterogeneity test, warranting careful consideration of the findings. In addition, survival bias may have a significant impact in our case, as the onset of T1DM occurs at a young age, and individuals who did not survive prior to conducting GWAS for either PD or AD may affect the quality of GWAS selected for use in MR analysis.

## Conclusions

In conclusion, this MR study unveiled a decreased genetic susceptibility to PD in individuals with T1DM, while no evidence of a genetic susceptibility between T1DM and AD was found. These findings suggest that T1DM may play a distinct role compared to T2D in the development of neurodegenerative diseases. Further research endeavors should be undertaken to unravel the underlying mechanisms that better elucidate this relationship.

### Supplementary Information

**Additional file 1: Figures S1–S6.** Heterogeneity and Horizontal pleiotropy analysis of IVW between AD and PD and T1DM.

## Data Availability

The GWAS summary data can be downloaded from GWAS (https://gwas.mrcieu.ac.uk/).
