# Supplementary material for: Causal relationships between type 1 diabetes mellitus and Alzheimer’s disease and Parkinson’s disease: a bidirectional two-sample Mendelian randomization study
Source: Eur J Med Res. 2024 Jan 16;29:53. doi: 10.1186/s40001-023-01628-z (PMC10790511; doi:10.1186/s40001-023-01628-z)
Supplement: Supplementary file 1 — Additional file 1: Figures S1–S6. Heterogeneity and Horizontal pleiotropy analysis of IVW between AD and PD and T1DM. [file 40001_2023_1628_MOESM1_ESM.zip › Supplementary material/S1.pdf]

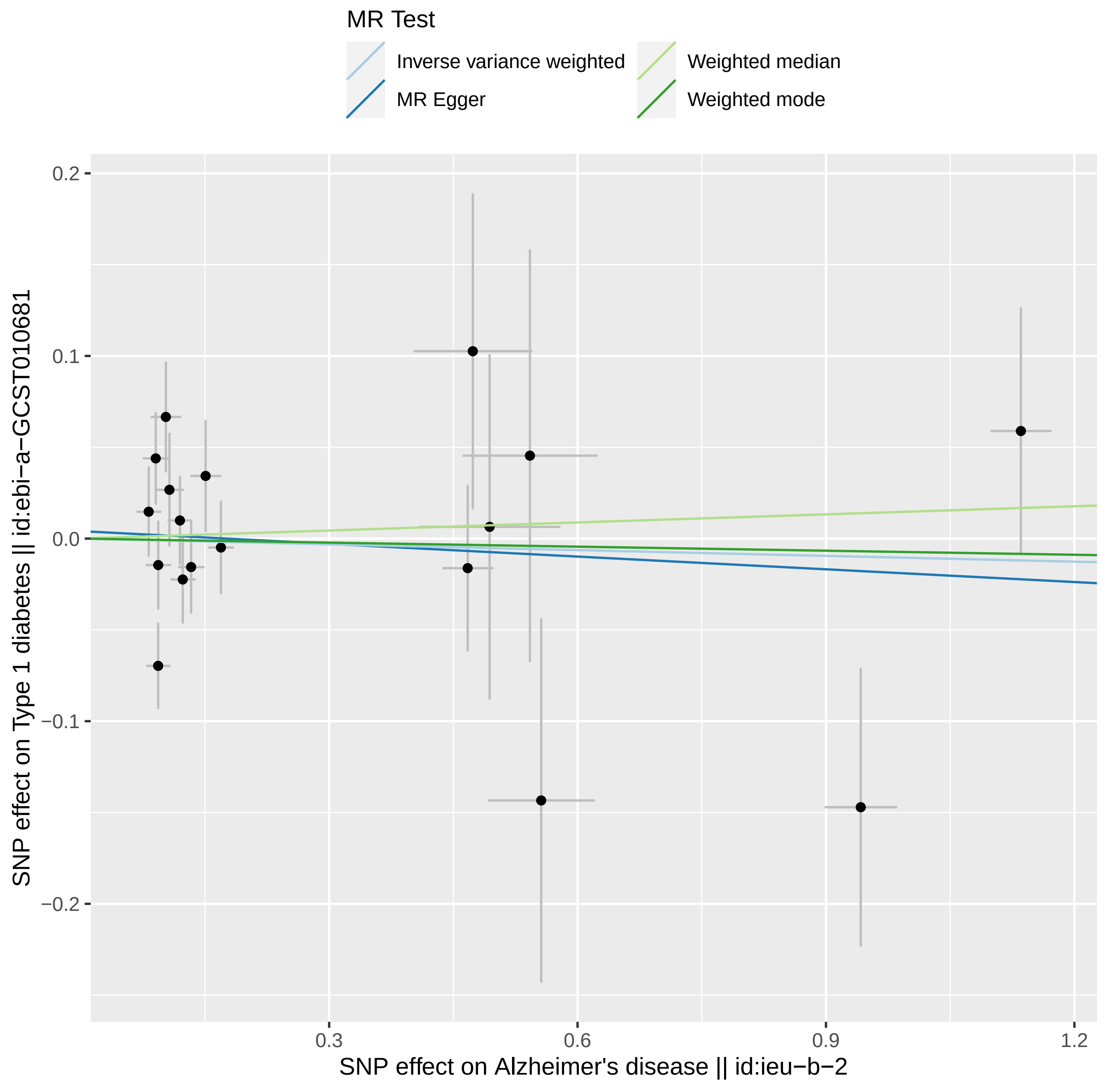

MR Test
Inverse variance weighted
MR Egger
Weighted median
Weighted mode
0.2
0.1
0.0
−0.1
−0.2
0.3
0.6
0.9
1.2
SNP effect on Type 1 diabetes || id:ebi−a−GCST010681
SNP effect on Alzheimer's disease || id:ieu−b−2
