# Supplementary figures and images for: Causal relationships between type 1 diabetes mellitus and Alzheimer’s disease and Parkinson’s disease: a bidirectional two-sample Mendelian randomization study
Source: Eur J Med Res. 2024 Jan 16;29:53. doi: 10.1186/s40001-023-01628-z (PMC10790511; doi:10.1186/s40001-023-01628-z)

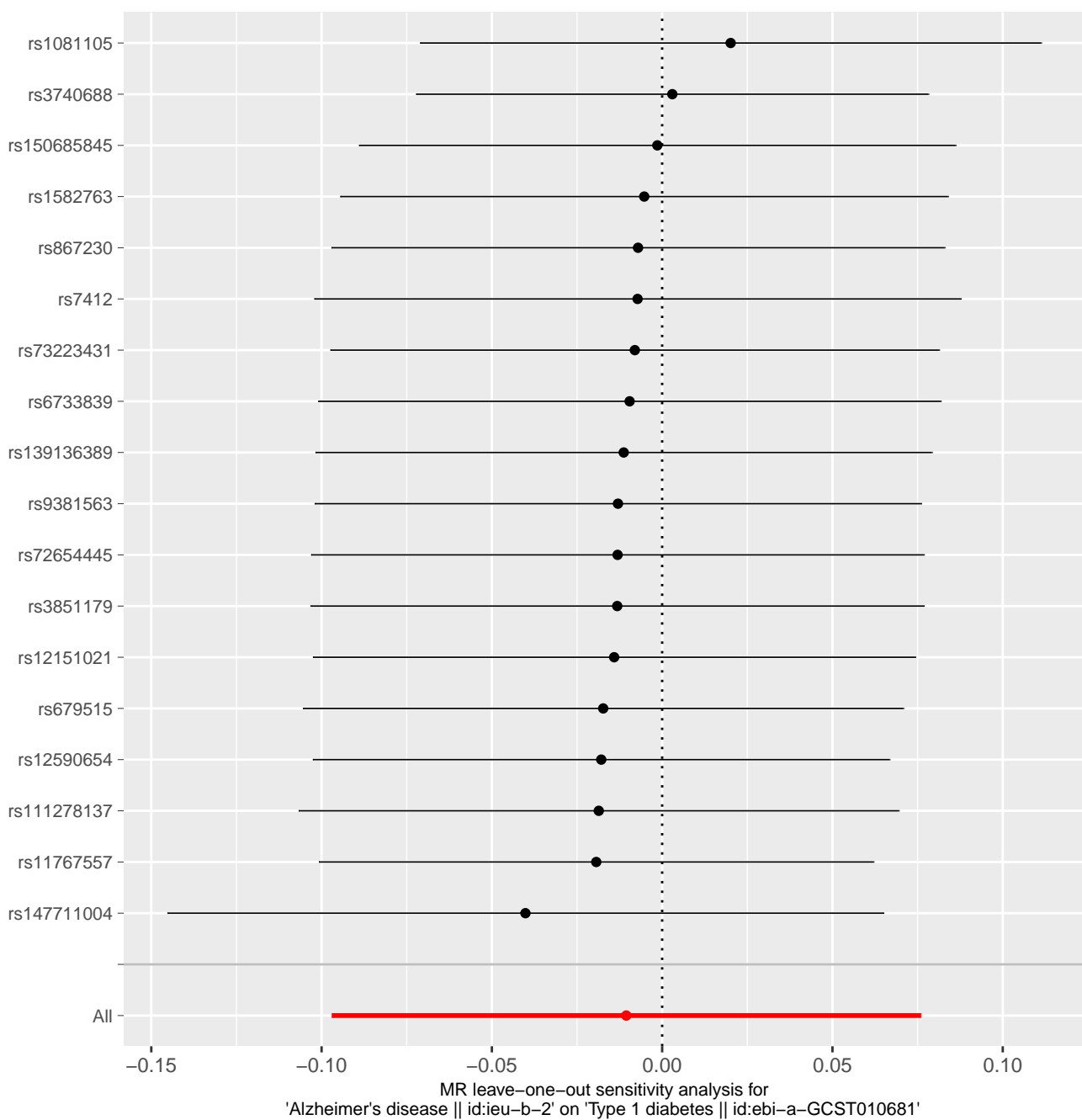

Supplement: Supplementary file 1 — Additional file 1: Figures S1–S6. Heterogeneity and Horizontal pleiotropy analysis of IVW between AD and PD and T1DM. [file 40001_2023_1628_MOESM1_ESM.zip › Supplementary material/S2.pdf]

# MR Method

- Inverse variance weighted
- MR Egger

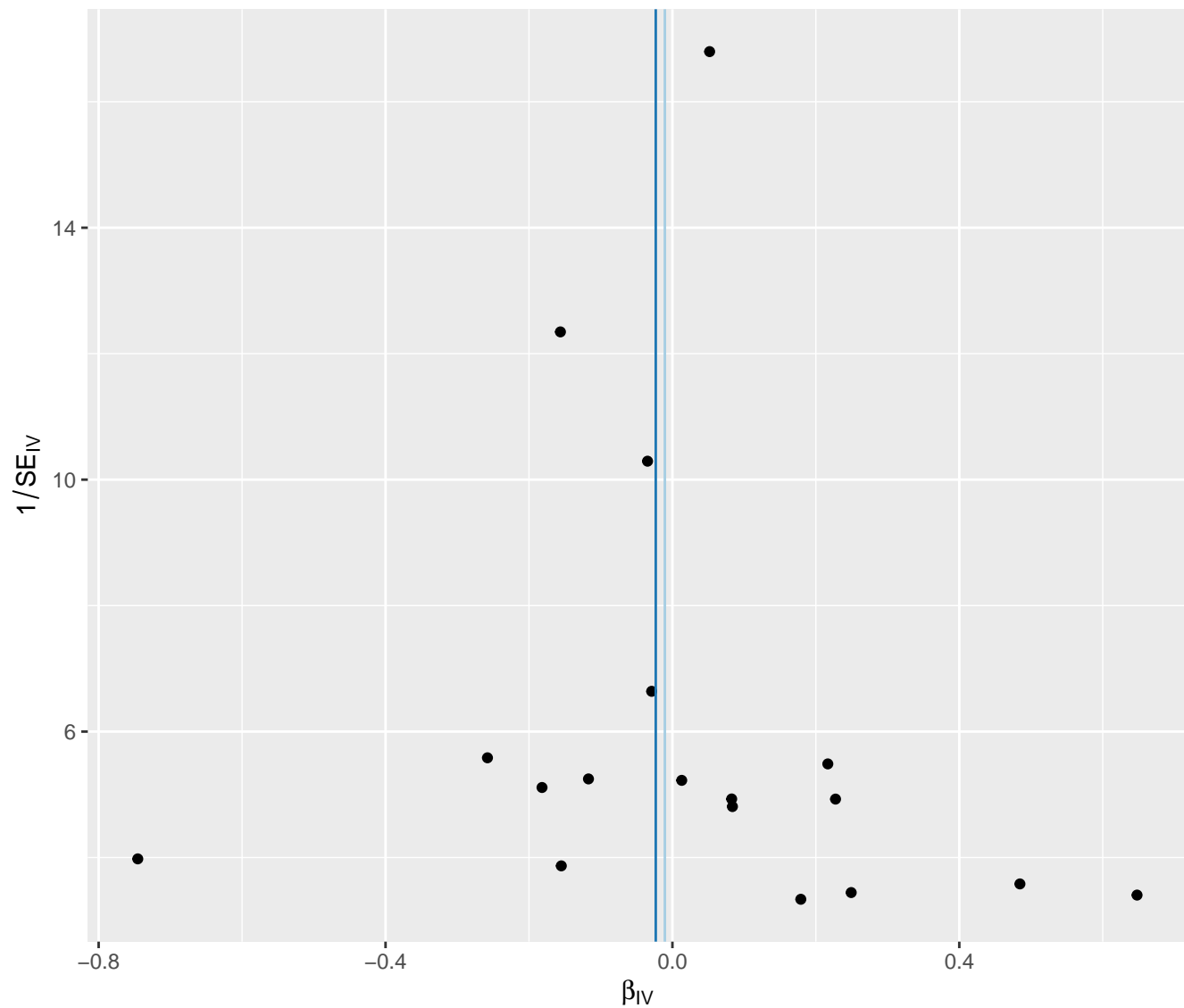

Supplement: Supplementary file 1 — Additional file 1: Figures S1–S6. Heterogeneity and Horizontal pleiotropy analysis of IVW between AD and PD and T1DM. [file 40001_2023_1628_MOESM1_ESM.zip › Supplementary material/S3.pdf]

# MR Test

- Inverse variance weighted
- MR Egger
- Weighted median
- Weighted mode

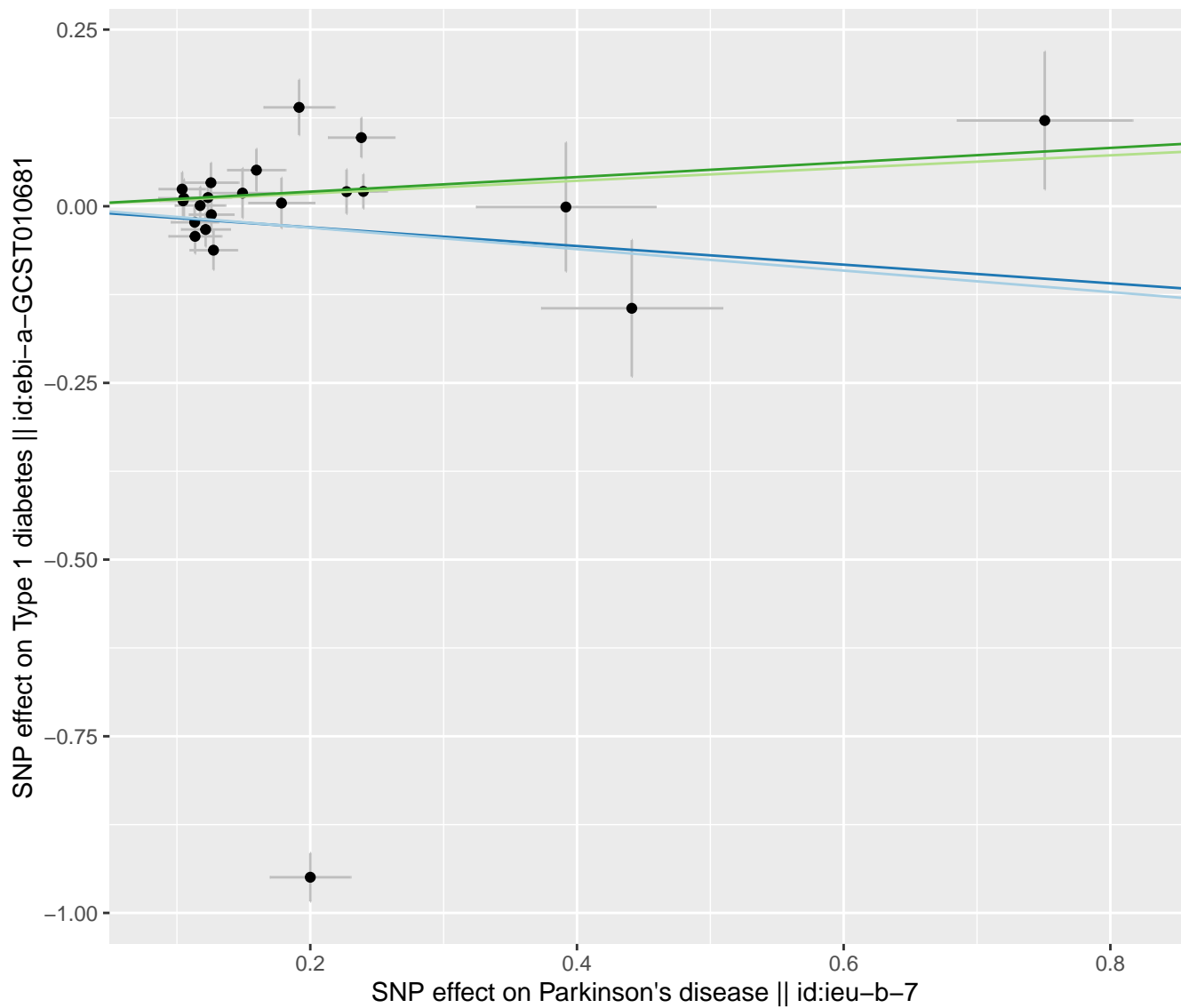

Supplement: Supplementary file 1 — Additional file 1: Figures S1–S6. Heterogeneity and Horizontal pleiotropy analysis of IVW between AD and PD and T1DM. [file 40001_2023_1628_MOESM1_ESM.zip › Supplementary material/S4.pdf]

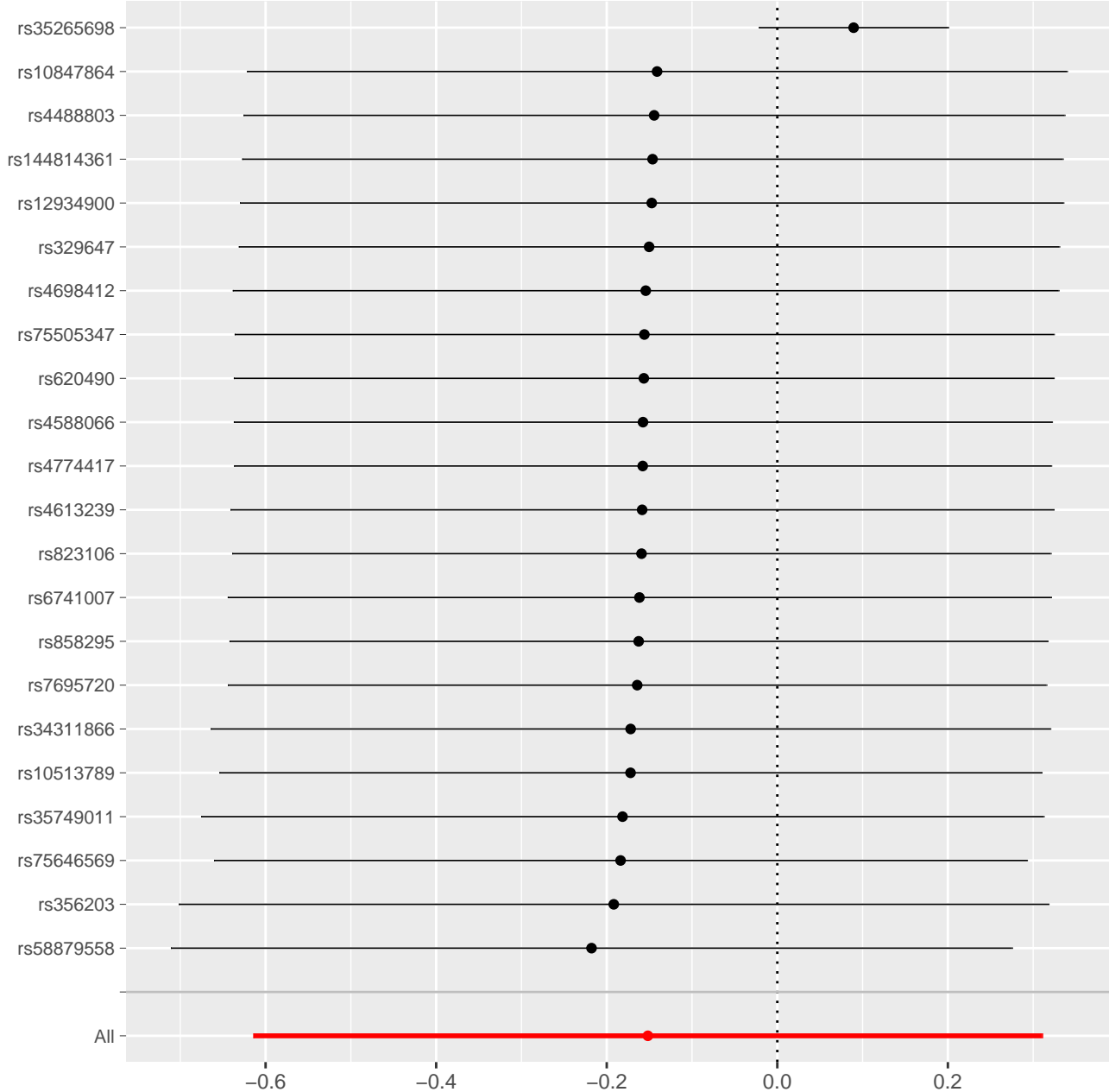

Supplement: Supplementary file 1 — Additional file 1: Figures S1–S6. Heterogeneity and Horizontal pleiotropy analysis of IVW between AD and PD and T1DM. [file 40001_2023_1628_MOESM1_ESM.zip › Supplementary material/S5.pdf]

# MR Method

- Inverse variance weighted
- MR Egger

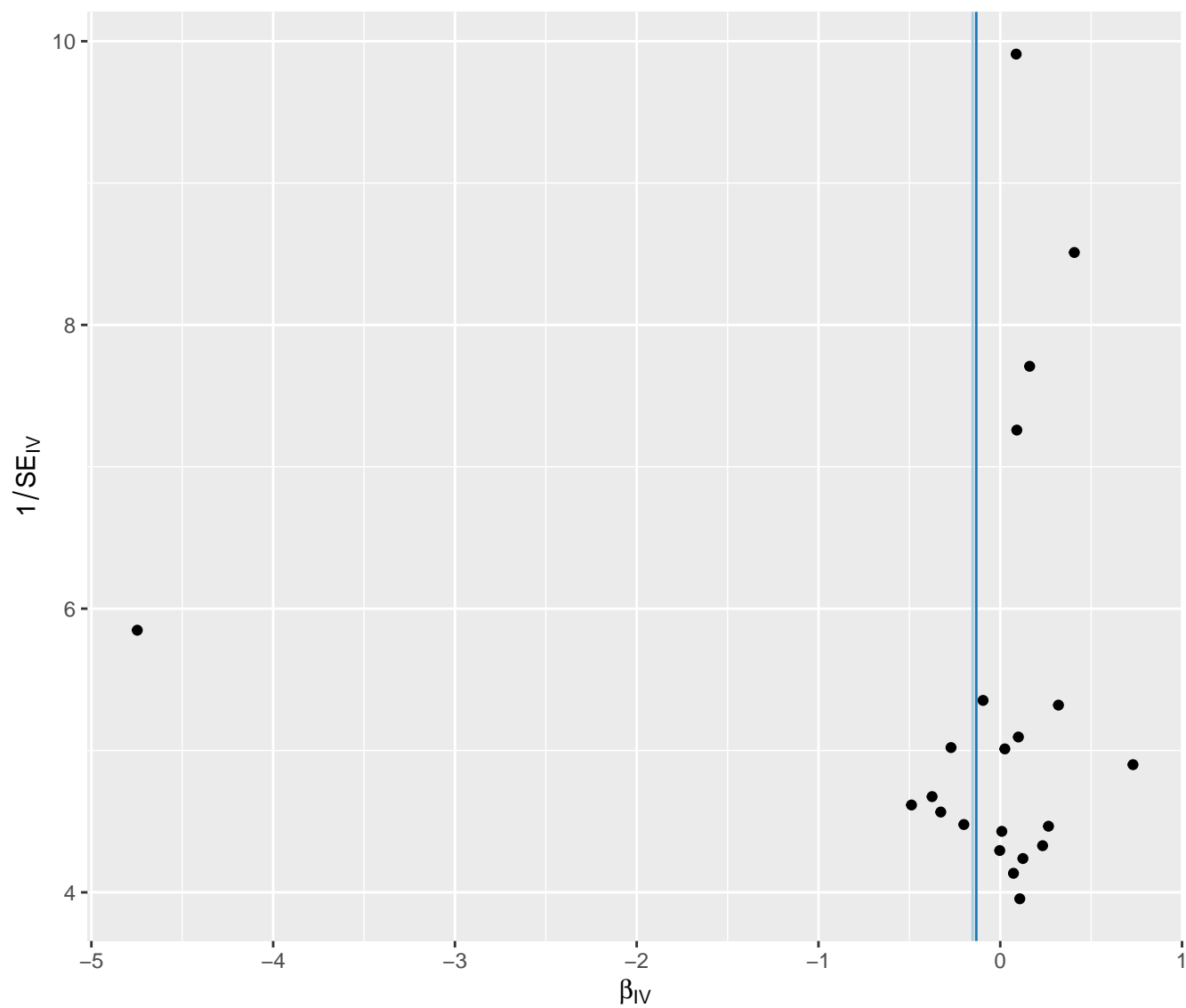

Supplement: Supplementary file 1 — Additional file 1: Figures S1–S6. Heterogeneity and Horizontal pleiotropy analysis of IVW between AD and PD and T1DM. [file 40001_2023_1628_MOESM1_ESM.zip › Supplementary material/S6.pdf]
